# Longitudinal association between leisure-time physical activity and vascular elasticity indices

**DOI:** 10.1186/s12872-021-01911-z

**Published:** 2021-02-16

**Authors:** Gábor Szaló, Margareta Hellgren, Matthew Allison, Lennart Råstam, Ulf Lindblad, Bledar Daka

**Affiliations:** 1 The Skaraborg Institute, Skövde, Sweden; 2 Primary Health Care, School of Public Health and Community Medicine, Institute of Medicine, Sahlgrenska Academy, University of Gothenburg, Gothenburg, Sweden; 3 University of California, San Diego, USA; 4 Department of Clinical Sciences, Lund University, Malmö, Sweden

**Keywords:** Vascular aging, Leisure-time physical activity, Vascular function, Small artery elasticity index, Arterial compliance

## Abstract

**Aim:**

We investigated the association between levels of leisure-time physical activity and vascular stiffness in a longitudinal observational study from a representative Swedish population.

**Method:**

A total of 2816 randomly selected individuals were examined at visit 1 (2002–2005, Men = 1400). After a mean follow-up of 9.7 ± 1.4 years, a representative sample of 1327 of the original participants were re-examined at visit 2. After excluding subjects with hypertension at baseline, 761 participants were included in the longitudinal analyses. Leisure-time physical (LTPA) activity was self-reported and dichotomized as high or low (level 3, 4 and level 1, 2, respectively). Large Arterial Elasticity Index (LAEI) and Small Arterial Elasticity Index (SAEI) were measured using the HDI/Pulse Wave™ CR2000. Multivariable general linear models were used to investigate the differences in changes SAEI and LAEI based on LTPA levels.

**Results:**

At visit 1, and after adjustment for possible confounders, participants in the high LTPA group had better small artery elasticity (SAEI) (SAEI in low-level LTPA: 7.89 ± 0.11, SAEI in high-level LTPA: 8.32 ± 0.15, ΔSAEI: 0.42, CI: 0.07–0.78; *p* = 0.020). SAEI decreased between the two assessments (Visit 1: SAEI 8.01 ± 3.37 ml/mmHg; Δ SAEI: 1.4, CI 1.2–1.6, *p* < 0.001). Participants with a higher LTPA at visit 1 had significantly better SAEI at visit 2 (ΔSAEI: 0.44, CI 0.03–0.85, *p* = 0.037). No significant associations were observed between LAEI and LTPA after adjustments.

**Conclusions:**

High LTPA predicted higher small arterial compliance at visit 2 suggesting that positive effects of LTPA on arterial elasticity persists over time.

## Introduction

One of the most important goals in primary care is to identify individuals with increased cardiovascular risk. High blood pressure is the most robust risk factor for cardiovascular disease [1, 2]. Blood pressure is considered to contain a steady component, mean blood pressure, and a pulsatile component, the pulse pressure. Hemodynamic research has shifted away from studying steady flow toward investigating pulsatile flow to include measurement of arterial elasticity [3]. Measures of arterial elasticity provide extra prognostic information beyond arterial blood pressure measurement [4] and can be used both for early detection of vascular disease and identification of increased risk for cardiovascular diseases (CVD) [5].

Early atherosclerosis is characterized by endothelial dysfunction followed by structural changes [5] including increased arterial stiffness, particularly in the smaller arteries [6]. In this regard, arterial elasticity largely depends on the ratio of elastin to collagen in arterial walls. The main changes are represented by degeneration of elastin material and an increase in collagenous material, often accompanied by calcium deposition in the ground substance and degenerated elastic fibres [7]. Vascular elasticity can be measured by diastolic pulse contour analysis of the arterial waveform which is a reproducible and reliable method [8, 9]. Duprez et al. evaluated systolic and diastolic pulse wave analysis and found that both of these indices correlated with the Framingham Risk Score [3]. In the Multi-Ethnic Study (MESA) Small Artery Elasticity Index (SAEI) was significantly associated with coronary disease, stroke, and heart failure [4].

In cross-sectional studies, it has been observed that individuals with low levels of physical activity have increased vessel stiffness, compared to active individuals [10–12]. According to a systematic review, aerobic exercise significantly increases vascular elasticity and the effect was better at higher exercise intensity and in participants with greater arterial stiffness at baseline [13]. To our best knowledge, there are only a few observations on how physical activity affects changes in vascular elasticity [11, 14, 15] and studies in representative populations and long-term follow-up are still missing. Thus, this study aimed to investigate the relationship between physical activity and vascular elasticity with a specific focus on whether low levels of physical activity are associated with the development of vascular stiffness in a longitudinal observational study from a representative Swedish population without hypertension.

## Method

### Study population

**Fig. 1:**
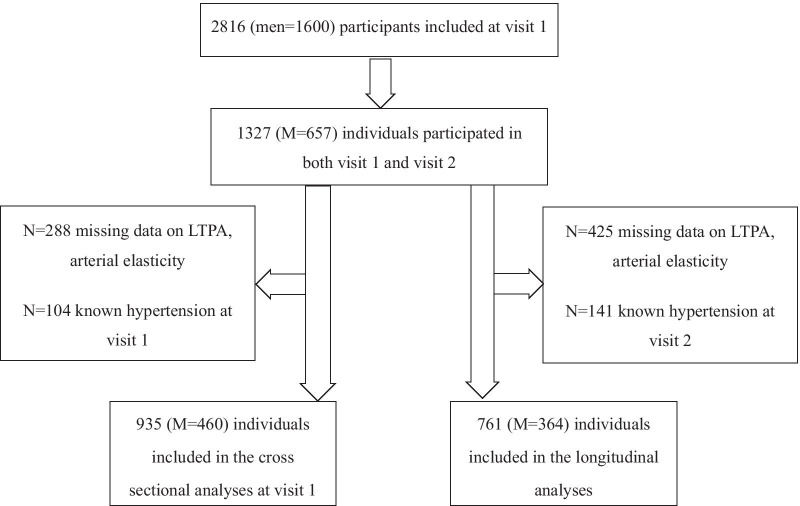
Study population

Details on Skaraborg population study have been described in detail previously [16]. In brief, it is a longitudinal observational study based on representative participants from Vara and Skövde municipalities in south-western Sweden. Between 2002 and 2005, a total of 2816 randomly selected individuals were examined (Men = 1400) for early signs off cardiovascular disease and risk factors for the development of CVD. After 10 years (9.7 years), and between 2012 and 2014, a representative sample of 1327 people were re-examined according to the same protocol as at visit 1 (Fig. 1). Two hundred eighty-eight participants do not have data on (Leisure Time Physical Activity) LTPA or arterial elasticity at visit 1, the corresponding number is 425 at visit 2. The number of participants with known hypertension was 104 at visit 1, and 141 at visit 2, respectively. Hypertensive medication can interfere with the elasticity indices significantly thus we chose to exclude participants with known hypertension at any of the visits. As such, in the cross-sectional analysis, 935 participants were included, while 761 participants were included in the longitudinal analysis at both visit 1 and 2 (Fig. 1).

### Measurements

Detailed information on the medical history including heart failure, atrial fibrillation, myocardial infarction, stroke, undergoing coronary heart surgery, as well as medication psychosocial and lifestyle was collected using validated questionnaires. All participants were asked alcohol [17] and smoking habits using validated questionnaires.

Body height and weight were measured in light clothes and no shoes to the nearest 1 cm and 0.1 kg, while waist- and hip circumferences were measured between the lowest rib margin and the iliac crest and at the largest circumference between waist and thighs, respectively. The blood pressure was measured to the closest 2 mmHg in the right arm after a five minutes rest in a supine position [16, 18]. Hypertension was defined based on Blood Pressure Management JNC-7 [19].

All participants underwent an oral glucose tolerance test (OGTT), whereby fasting and 2 h glucose values were obtained. Diabetes diagnosis was based on the 1999 WHO recommendation [20]. Fasting plasma insulin was analyzed using an enzyme-linked immunosorbent assay (ELISA) with < 0.3% cross-reactivity for proinsulin. Insulin resistance by HOMA-IR was calculated with the formula below [21]: fasting insulin (micro IU/L) x fasting glucose (nmol/L)/22.5. Assays for LDL cholesterol, triglyceride, C-reactive protein (CRP) and creatinine were conducted.

### Leisure-time physical activity

The level of physical activity was estimated using a question about Leisure Time Physical Activity (LTPA) which has been validated in several previous studies [22–24]. All subjects were asked for their LTPA in the last 2 weeks. This scale has four levels based on four answer alternatives to the question “How much physical effort do you put yourself through in your leisure time?”.

1. *Inactive*, mostly physically inactive, and less strenuous LTPA (walking, cycling, gardening etc.) less than four hours a week (h/w);
2. *Moderate*, less strenuous LTPA > 4 h/w;
3. *Strenuous*, strenuous physical activity (jogging, swimming, tennis etc.) > 2 h/w and
4. *Highly strenuous,* including those who participate in competitions on a weekly basis

### Pulse wave analyses

Arterial compliance was measured based on diastolic pulse contour analysis of arterial waveform using the HDI/Pulse WaveTM CR2000 (Hypertension Diagnostics, Inc., Eagan, NY, USA). The tonometer was centered over the radial artery. The optimal radial artery waveform was obtained by non-invasive recording by applanation tonometry. This technique is based on a modified Windkessel model that gives an evaluation of the elasticity of the large conduit arteries (Large Arterial Elasticity Index, LAEI, C1) and the small microcirculatory arteries (Small Arterial Elasticity Index, SAEI, C2) [25, 26].

### Statistics

Data were analyzed using SPSS 26. LTPA was grouped in 2 levels high and low. Subjects inactive and moderately active was classified as low LTPA and the others as high LTPA (high LTPA = level 3 and 4, low LTPA = level 1 and 2). General linear models were used to investigate the differences between these two groups in large and small elasticity indices at visit 1 and then changes over the two study visits. Adjustments for possible confounding were based on theoretical models, stepwise with each model building upon the previous one. In model, 1 we adjusted for age, sex, heart rate and systolic blood pressure, while in model 2 we adjusted for the variables in Model 1 plus diabetes mellitus, HOMA-IR, cardiovascular morbidity and LDL cholesterol and model 3 included model 2 variables plus alcohol consumption and smoking. In both LTPA-groups, we examined how SAEI changes during the follow up time. Predictive models were built to investigate whether the level of LTPA at visit 1 was associated with levels of LAEI and SAEI at visit 2. Two-way interaction terms showed no differences between men and women in the association between LTPA and SAEI (*p* = 0.596 at visit 1). Thus, we choose to investigate men and women together and to adjust the analysis for gender.

## Results

The average follow-up time was 9.7 ± 1.4 years. The average age of the study population at visit 1 was 46.7 ± 10 years (47.4 for men, 46.2 for women), while being 54.6 ± 10 years old at visit 2 (54.8 for men and 54.5 for women). In all, 610 participants reported low and 325 high LTPA at visit 1. Out of them, 64 persons (6.8%) were inactive (level 1), 546 (58.4%) were moderately active (level 2), 294 (31.4%) were active (level 3) and 31 (3.3%) were very active (level 4). Based on previous experience [27] and in the low number of subjects in the highest and the lowest level we than dichotomized into 2 groups. Correspondingly, 454 participants reported low and 307 high LTPA at visit 2.

Participants with high LTPA at both visits had lower prevalence in T2DM and hypertension than those with low LTPA. There was a significant difference between the groups of high- and low-LTPA in terms of the mean value for heart rate, and metabolic parameters such as fasting glucose, HOMA-IR, triglycerides (Table 1). No significant differences between the group with high and low LTPA were observed in LDL cholesterol levels and cardiovascular morbidity (Table 1).

**Table 1:** Characteristics of the participants for visit 1

	All n = 935	Low LTPA n = 610	High LTPA n = 325	*p*
Age, years (SD)	46.7 (10)	47.5 (11)	44.4 (10)	0.004*
Gender, male (%)	460 (49)	281 (61)	179 (39)	0.009*
BMI, kg/m^2^ (SD)	26.4 (4)	26.8 (4)	25.5 (3)	< 0.001*
SBP, mmHg (SD)	119 (14)	119 (15)	117 (14)	0.055
DBP, mmHg (SD)	69 (10)	69 (10)	69 (9)	0.993
Pulse, bpm (SD)	63 (8)	64 (8)	62 (8)	< 0.001*
Fasting glucose, mmol/l (SD)	5.3 (0.7)	5.3 (0.7)	5.2 (0.6)	0.009*
HOMA-IR median (IQR)	1.16 (0.81–1.78)	1.26 (0.88–1.89)	1.09 (0.7–1.55)	< 0.001*
Triglyceride, mmol/l median (IQR)	1.05 (0.77–1.46)	1.08 (0.79–1.55)	1.01 (0.68–1.34)	< 0.001*
LDL cholesterol, mmol/l (SD)	3.3 (0.9)	3.3 (0.9)	3.2 (0.9)	0.093
CRP, mg/l median (IQR)	1.3 (0.7–2.4)	1.4 (0.8–2.6)	1 (0.6–1.9)	0.004*
Diagnosis of hypertension n (%)	41 (4.4)	30 (4.9)	11 (3.4)	0.276
Diagnosis of DM n (%)	20 (2.1)	15 (2.5)	5 (1.5)	0.355
Cardiovascular morbidity n (%)	14 (1.5)	11 (1.8)	3 (0.9)	0.293
Current smoker n (%)	151 (16)	106 (17)	45 (14)	0.163
Alcohol n [g/week] median (IQR)	25.6 (9.3–61.5)	24.4 (6.3–59.5)	30.1 (11.5–67.6)	0.805
SAEI mL/mmHg (SD)	8.01 (3.37)	7.65 (3.35)	8.68 (3.32)	< 0.001*
LAEI mL/mmHg (SD)	16.65 (4,95)	16.24 (4.92)	17.41 (4.92)	0.001*

LTPA: Leisure-Time Physical Activity, p: significance between low and high LTPA, BMI: Body Mass Index, SBP: Systolic Blood Pressure, DBP: Diastolic Blood Pressure, HOMA-IR: Homeostatic Model Assessment for Insulin Resistance, LDL: Low-Density Lipoprotein, DM: Diabetes Mellitus, SAEI: Small Arterial Elasticity Index, LAEI: Large Arterial Elasticity Index, IQR: interquartile range

Overall, artery elasticity measured by SAEI and LAEI decreased during the observational time (Visit 1: SAEI 8.01 ± 3.37 ml/mmHg, LAEI 16.65 ± 4.95; Δ SAEI: 1.41, CI 1.19–1.61, *p* < 0.001: Δ LAEI: 1.64, CI 1.29–1.98, *p* < 0.001). At visit 1, SAEI was 7.65 ± 3.35 ml/mmHg in subjects with low-LTPA and 8.68 ± 3.32 ml/mmHg in subjects with high-LTPA (*p* < 0.001), while at visit 2, SAEI was 6.64 ± 3.17 ml/mmHg and 7.71 ± 3.29 ml/mmHg (*p* < 0.001), respectively. The same trend was observed in case of LAEI. Specifically, LAEI was 16.24 ± 4.92 in individuals with a low level of LTPA and 17.41 ± 4.92 in individuals with a high level of LTPA at visit 1 (*p* = 0.001), and then 15.16 ± 5.04 and 16.30 ± 4.90 respectively at visit 2 (*p* < 0.001).

Participants with a high level of LTPA had significantly higher SAEI at visit 1 (Crude model) (SAEI in low-level LTPA: 7.65 ± 0.14, SAEI in high-level LTPA: 8.68 ± 0.19 Δ SAEI: 1.03, CI 0.6–1.15, *p* < 0.001) and this difference remained significant after adjustment for possible confounding according to Model 3 (SAEI in low-level LTPA: 7.89 ± 0.11, SAEI in high-level LTPA: 8.32 ± 0.15, ΔSAEI: 0.42, CI 0.07–0.78; *p* = 0.020) (Table 2). In model 3 after adjusting for BMI instead of HOMA-IR, similar results were found (Δ SAEI: 0.50, CI: 0.15–0.86; *p* = 0.050). No significant association were observed between large vessels elasticity and LTPA after adjustments for Model 1 (*p* = 0.690).

**Table 2:** Association between the level of leisure-time physical activity and small artery elasticity index at visit one

N = 935		
Mean difference	Confidence interval (CI)	*p* value
Crude data		
1.03 ml/mmHg	0.58–1.48	< 0.001
Adjusted for age, sex, heart rate and systolic blood pressure		
0.40 ml/mmHg	0.05–0.75	0.024
Adjusted as above and for diabetes mellitus, HOMA-IR, LDL cholesterol and cardiovascular comorbidity		
0.47 ml/mmHg	0.12–0.83	0.009
Adjusted as above + alcohol consumption and smoking		
0.42 ml/mmHg	0.07–0.78	0.020

All patients are included who filled in scales about physical activity with baseline and have data for pulse wave analysis with baseline. Patients with known hypertension are excluded

*HOMA-IR* Homeostatic Model Assessment for Insulin Resistance

**Fig. 2:**
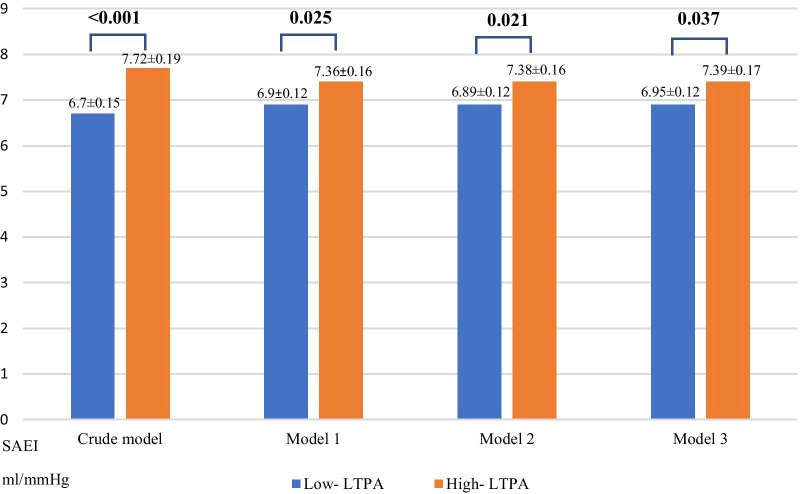
The association between leisure-time physical activities at visit one and small artery elasticity index at visit two

During the follow up time we observed a decrease in SAEI by 11.9%. The decrease was slightly higher in the group with low LTPA (12.4%) when compared with the group with high LTPA (11.1%). However, these differences were not significant. In the longitudinal analyses, we observed that participants with a high LTPA at visit 1 had significantly higher vascular elasticity (SAEI) at visit 2 than subjects with low LTPA (ΔSAEI: 1.02, CI 0.05–1.5, *p* = 0.022) and these associations were strong and significant after adjustments for Model 3 (ΔSAEI: 0.44, CI 0.03–0.85, *p* = 0.037) (Fig. 2 and Table 3).

**Table 3:** The association between leisure-time physical activities at visit one and small artery elasticity index at visit two, Vara-Skövde cohort

N = 761		
Mean difference	Confidence interval (CI)	*p* value
Crude		
1.02 ml/mmHg	0.54–1.50	< 0.001
Adjusted for age, sex, heart rate and systolic blood pressure		
0.46 ml/mmHg	0.06–0.86	0.025
Adjusted as above + diabetes mellitus, HOMA-IR, LDL cholesterol and cardiovascular comorbidity		
0.48 ml/mmHg	0.07–0.89	0.021
Adjusted as above + alcohol consumption and smoking		
0.44 ml/mmHg	0.03–0.85	0.037

All patients are included who filled in scales about physical activity with baseline and have data for pulse wave analysis with baseline and follow up. Patients with known hypertension (baseline and follow-up) are excluded

*HOMA-IR* Homeostatic Model Assessment for Insulin Resistance

We investigated if the aforementioned associations were different by sex. At both visits, we found that differences between men and women in combination with differences between LTPA levels and SAEI were not significant (*p* = 0.596 at visit 1, *p* = 0.307 at visit 2).

Equivalent analyses regarding LAEI did not show significance (*p* = 0.286 at visit 1, *p* = 0.266 at visit 2).

## Discussion

### Main findings

In this study, we showed a strong and independent association between leisure-time physical activities and small artery elasticity index independent of possible confounders both at baseline and approximately 10 years later. The association between SAEI and LTPA was strong and significant even after adjusting in the full model. These results indicate potential salutary effects of exercise on SAEI that may be persistent longitudinally.

Saladini et al. observed the effect of physical activity in 151 subjects with hypertension during a period of 6 years [14]. In line with our findings, they also found that physical activity predicted better SAEI. Moreover, in the Whitehall II study [15], the follow-up time was 5 years and found that moderate-to-vigorous, but not mild, activity was associated with slower progression of aortic stiffness over time. Notably, we were able to include HOMA-IR, diabetes mellitus, cardiovascular morbidity, LDL cholesterol and alcohol consumption as covariates in the models. Our study confirmed these findings and expanded them even in the non-hypertensive population. To our knowledge this is the first study to investigate the association between reported leisure time physical activities and small artery elasticity index in a middle-aged representative cohort of men and women without hypertension.

Physical activity has many benefits both with traditional cardiovascular risk factors and with mortality [28, 29]. In cross-sectional observations, higher artery elasticity is associated with being physically active [10, 12, 13, 30]. In line with these observations, our study found that all indices were significantly better with higher LTPA level at both visits before adjustments.

Small artery elasticity index (SAEI) seems to be strongly associated with changes in the artery wall associated with hypertension [14], diabetes mellitus, and atherosclerosis compared to LAEI [6]. Similarly, it is strongly associated with chronic heart disease, stroke and heart failure [4]. In our study, difference in LTPA levels seems to have a stronger effect on SAEI than LAEI. The stronger association between LTPA levels and SAEI compared with LAEI might depend on that the development of the atherosclerotic disease changes in the endothelial function before structural changes [5] including increased arterial stiffness, particularly in the smaller arteries [6]. In fact, physical activity reduces the plasma endothelin concentration the endothelin mediated vascular tone and thus reduces arterial stiffness [31]. Moreover, physical activity improves endothelial function by up-regulating endothelial nitric oxide synthase (eNOS) protein expression [32]]. Regular physical activity has long-term anti-inflammatory effects and improves metabolic control. It is suggested this occurs by skeletal muscle serving as an endocrine organ through a production and secretion of myokines in response to exercise. These mechanisms affect many organs and metabolic pathways, including endothelial cell function [33].

Arterial elasticity indices change over time because of structural modifications in the blood vessels wall like the degeneration of elastin material and an increase in collagenous material and in-ground substance [7]. The factors which can affect the stiffening processes are many, but want to underline ageing, blood pressure, blood glucose, lipids, diseases (hypertension, diabetes mellitus type 1 and 2, coronary artery diseases, heart failure, stroke), medication, lifestyle (smoking, alcohol consumption, physical activity). We have adjusted for these possible confounders in our statistical analysis and found that high level of leisure-time physical activity was still associated with small artery elasticity index suggesting an independent association with these variables.

### Strengths and limitations

All measurements were computed by the same trained staff using a strict protocol providing a strong internal validity in the measurements. The large variety of information and the variables provided by the study permitted the testing of different models. In this study, the inclusion of both men and women made it possible to investigate possible gender differences in the associations and interaction analyses. Even if these differences in SAEI and LAEI between groups with low respective high LTPA are small numerically, these differences are significant at group level and it is consistent with the expected effect of physical activity. A high participation rate at both visits gave higher power to our analyses. A weakness of the study is the use of self-reported physical activity, only providing a subjective estimation of the leisure time physical activities. This scale is however validated and seems to reflect with good approximation the physical activities. Our study was observational without randomization, thereby there is a risk for residual confounding.

### Clinical implications

Cardiovascular prevention is one of the main tasks in primary care. Early introduction of lifestyle change especially physical activity might be important to reduce cardiovascular risk through positive effects on vascular elasticity. Public health strategies introducing leisure-time physical activities early and helping the population to maintain it during life should be effective in maintaining good vascular health, reducing the incidence of hypertension and cardiovascular disease.

## Conclusions

We found a positive salutary effect on artery elasticity by physical activity. A higher level of physical activity (LTPA) predicted a higher SAEI in this cohort. Our result suggest that physical activity may inhibit the age-related decline in artery elasticity and arterial compliance.

## Data Availability

The datasets used and analyzed during the current study available from the corresponding author on request.
